# Supplementary material for: Evolution and stabilization of subnanometric metal species in confined space by in situ TEM
Source: Nat Commun. 2018 Feb 8;9:574. doi: 10.1038/s41467-018-03012-6 (PMC5805776; doi:10.1038/s41467-018-03012-6)
Supplement: Supplementary file 1 — Supplementary Information [file 41467_2018_3012_MOESM1_ESM.pdf]

Supplementary Information

Evolution and stabilization of subnanometric metal species in confined space by *in situ* TEM

Liu *et al.*

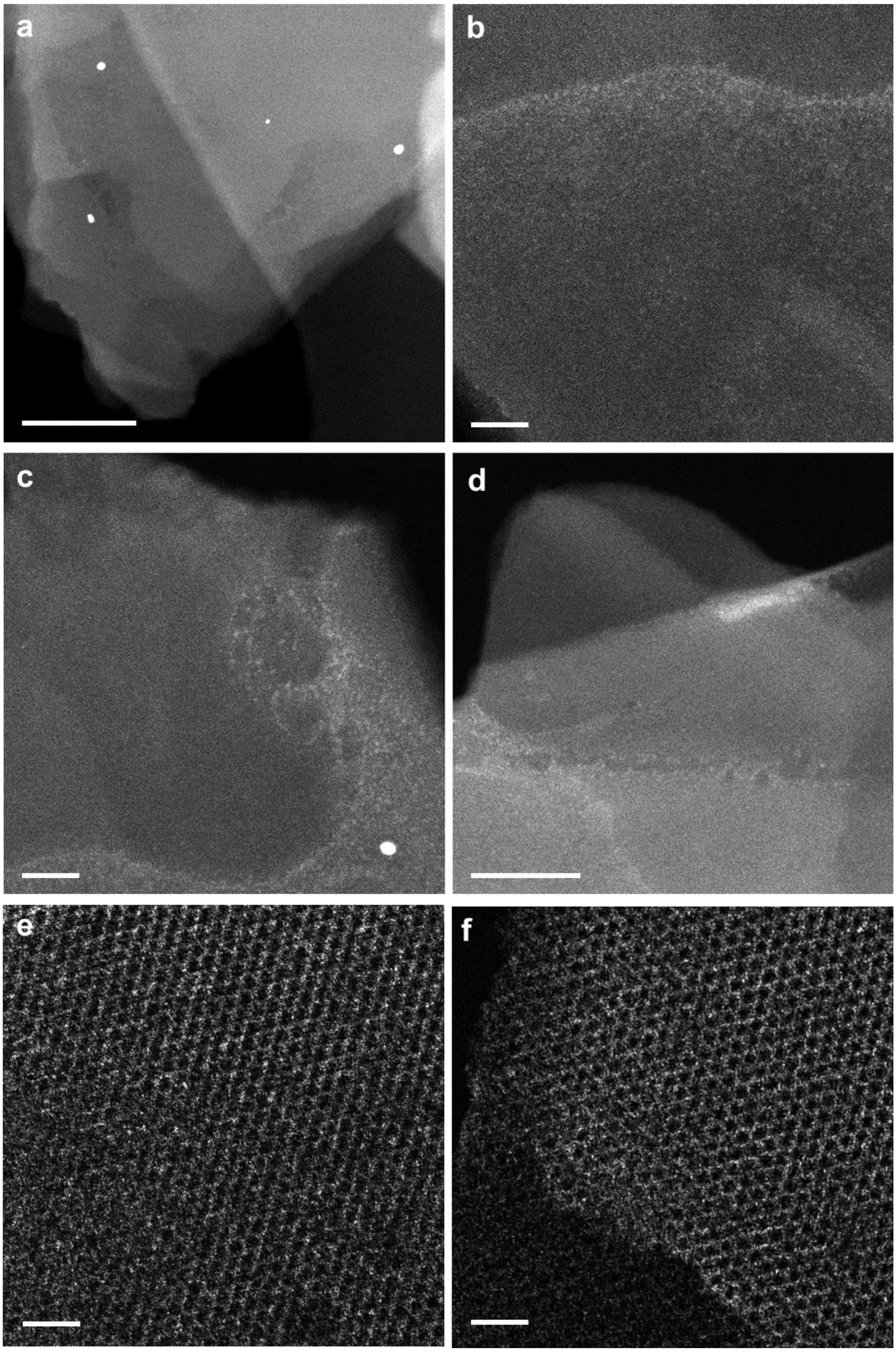

Supplementary Fig. 1 Morphological characterization of 0.17%Pt@MCM-22 sample after calcination in air at 550 °C. (a-d) STEM images of the 0.17%Pt@MCM-22 sample, showing the presence of subnanometric Pt clusters and a few Pt nanoparticles on the surface of MCM-22 zeolite. (e, f) High-resolution STEM images of 0.17%Pt@MCM-22 sample. As shown in these images, Pt single atoms as well as the pore structures of MCM-22 zeolite can be observed. Scale bar: (a) 100 nm, (b, c) 20 nm, (d) 50 nm and (e, f) 5 nm.

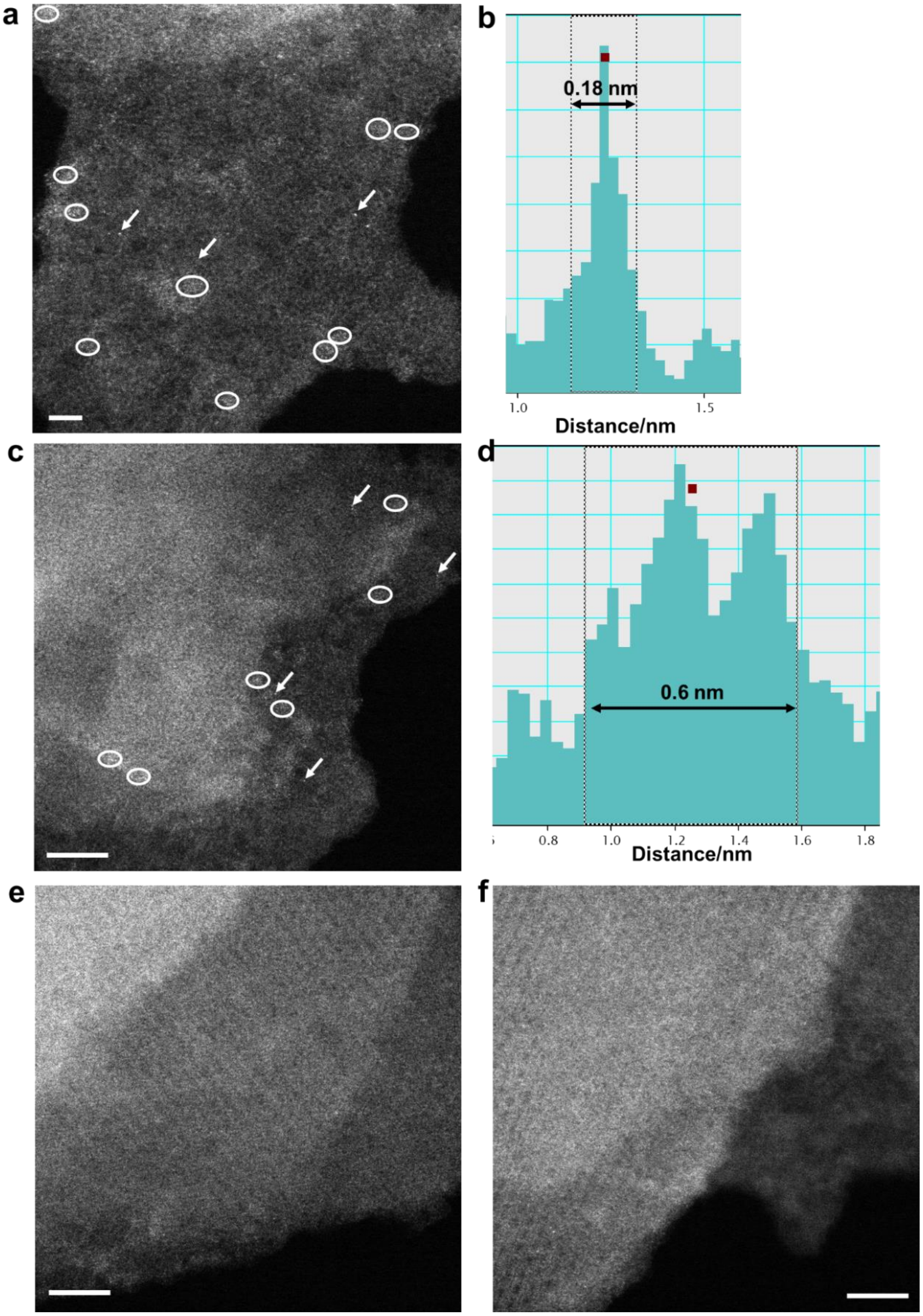

Supplementary Fig. 2 Identification of Pt single atoms and clusters in the 0.17%Pt@MCM-22 sample. As shown in (a), both Pt atoms (bright dots) and clusters (aggregates of several Pt atoms) can be seen. The intensity of a single Pt atom is displayed in (b). (c) Pt atoms and clusters in another area of the 0.17%Pt@MCM-22 sample. The intensity of a Pt cluster (~0.6 nm) is displayed in (d). (e, f) Two more representative STEM image of the 0.17%Pt@MCM-22 sample, showing the pore structure of MCM-22 and presence of subnanometric Pt species. Scale bar: (a) 2 nm, (c, e, f) 5 nm.

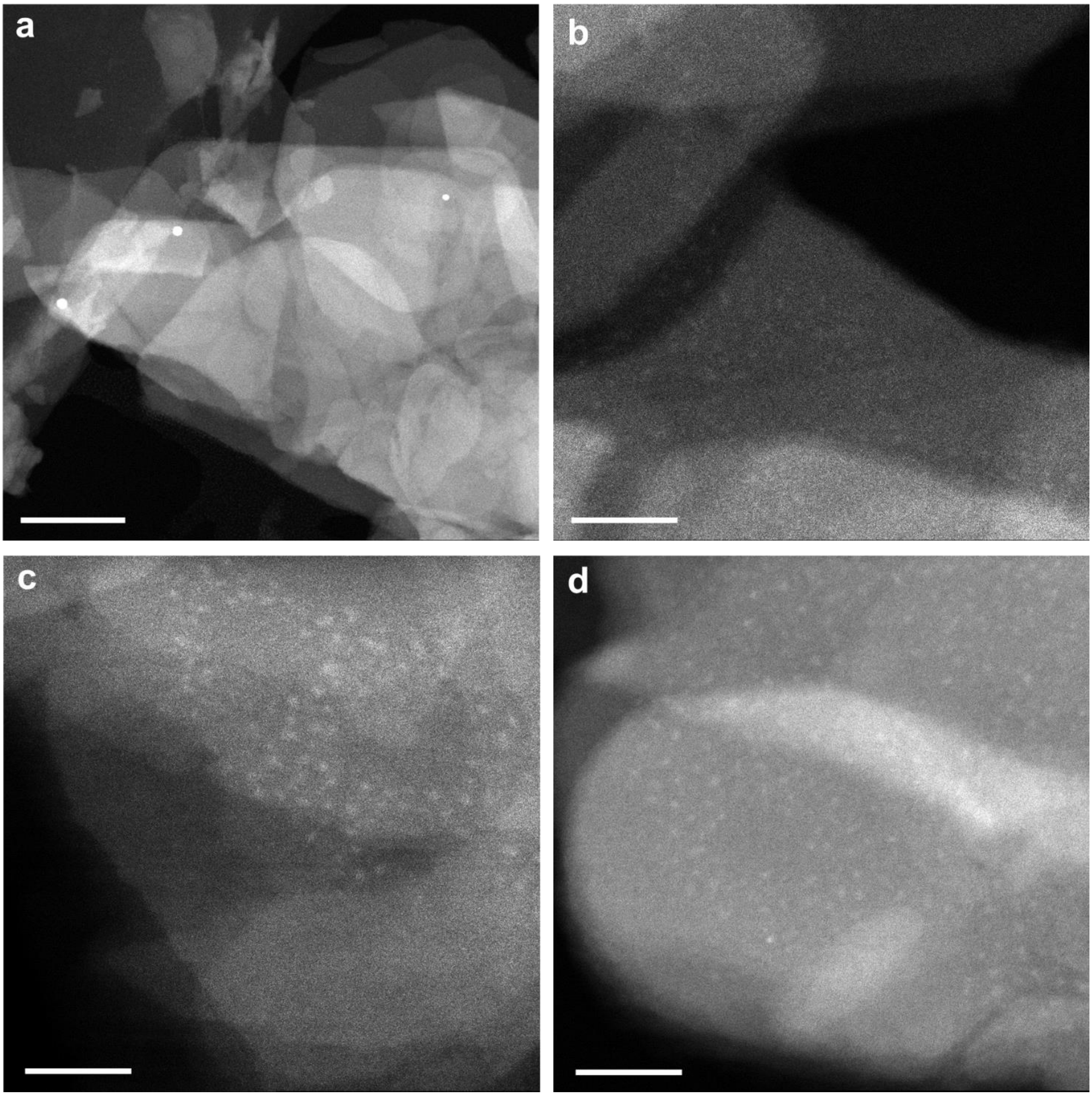

Supplementary Fig. 3 Morphological characterization of 0.17%Pt@MCM-22-200H_2. As shown in these images, Pt clusters can be observed in the sample after reduction by H_2 at 200 ºC. Scale bar: (a) 100 nm, (b-d) 20 nm.

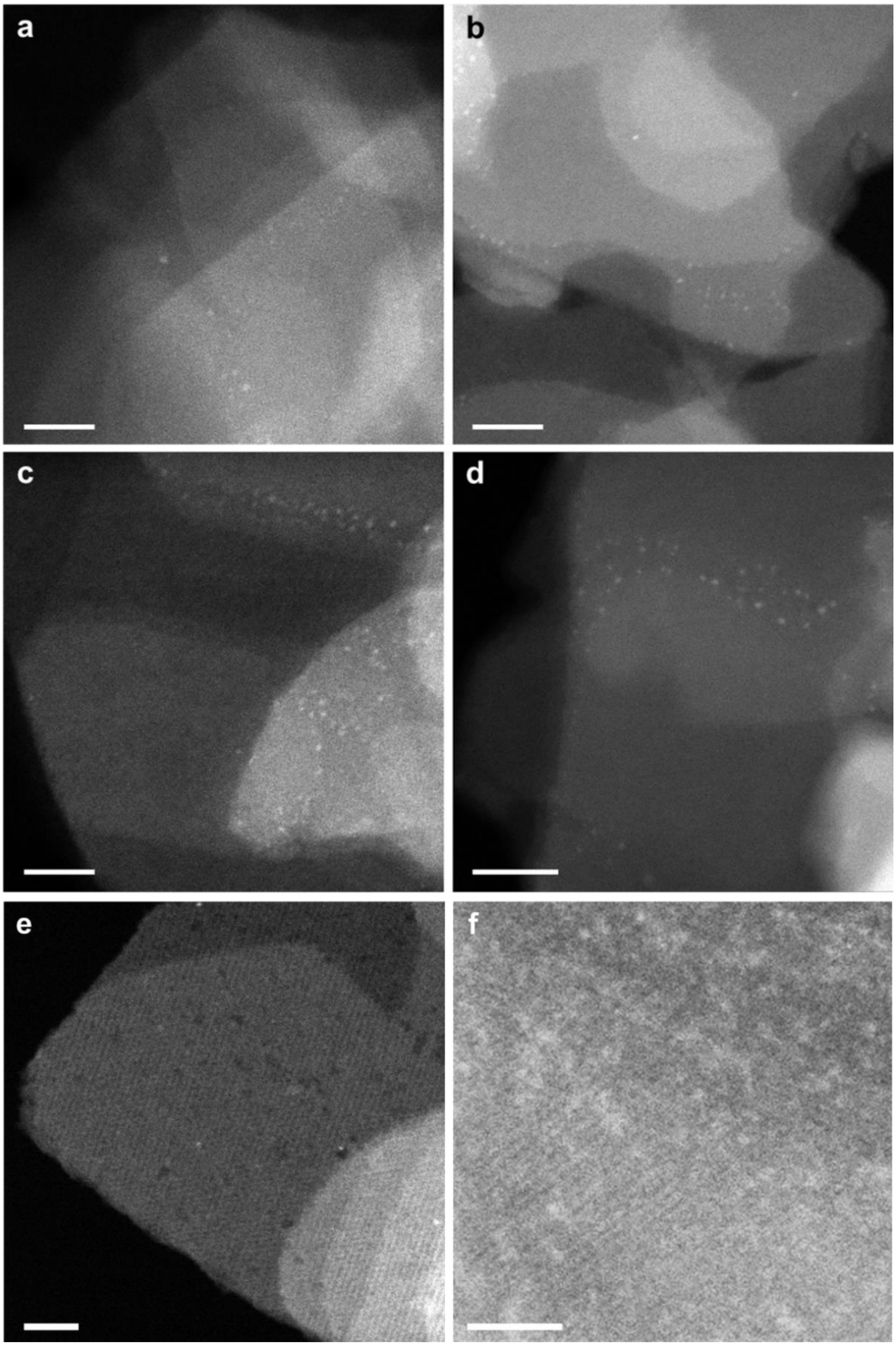

Supplementary Fig. 4 Morphological characterization of 0.17%Pt@MCM-22-300H$_2$ sample. (a-d) As shown in these images, part of the highly dispersed Pt species agglomerate into Pt clusters. The size of Pt clusters increases compared with the 0.17%Pt@MCM-22-200H$_2$ sample shown in **Supplementary Fig. 3**. In (e, f), the pore structures of MCM-22 can be observed. Moreover, the presence of subnanometric Pt species in this sample is also confirmed in these high-resolution STEM images. Scale bar: (a-d) 20 nm, (e, f) 10 nm.

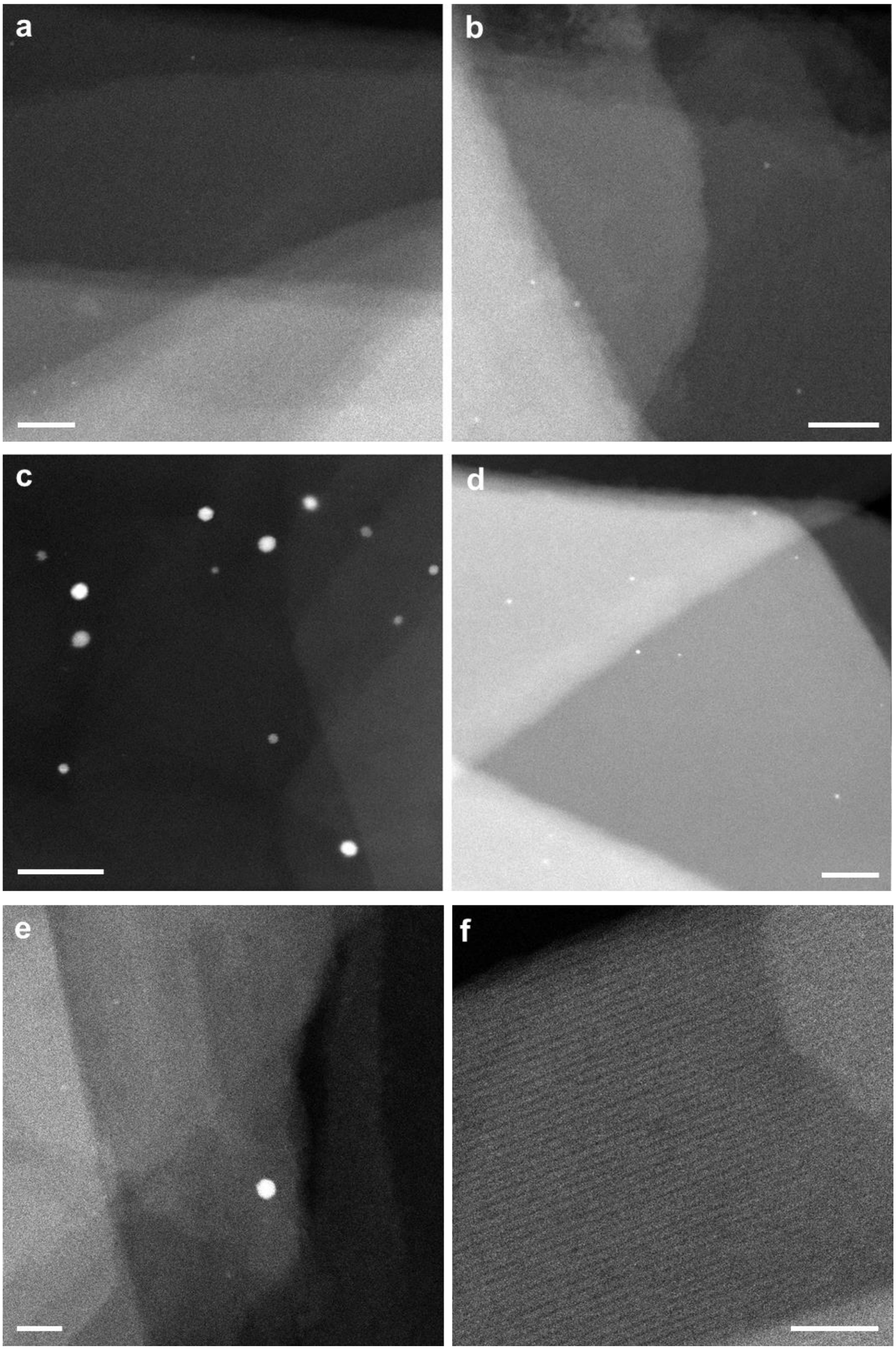

Supplementary Fig. 5 Morphological characterization of 0.17%Pt@MCM-22-400H_2 sample. (a-d) Pt clusters as well as some Pt nanoparticles around 1 nm can be observed in this sample. Besides, Pt nanoparticles from the agglomeration of Pt species on the surface of MCM-22 crystallites can be observed. (e, f) Pt single atoms are not observed in the high-resolution STEM images. Scale bar: (a, b, d) 20 nm, (c) 50 nm, (e, f) 10 nm.

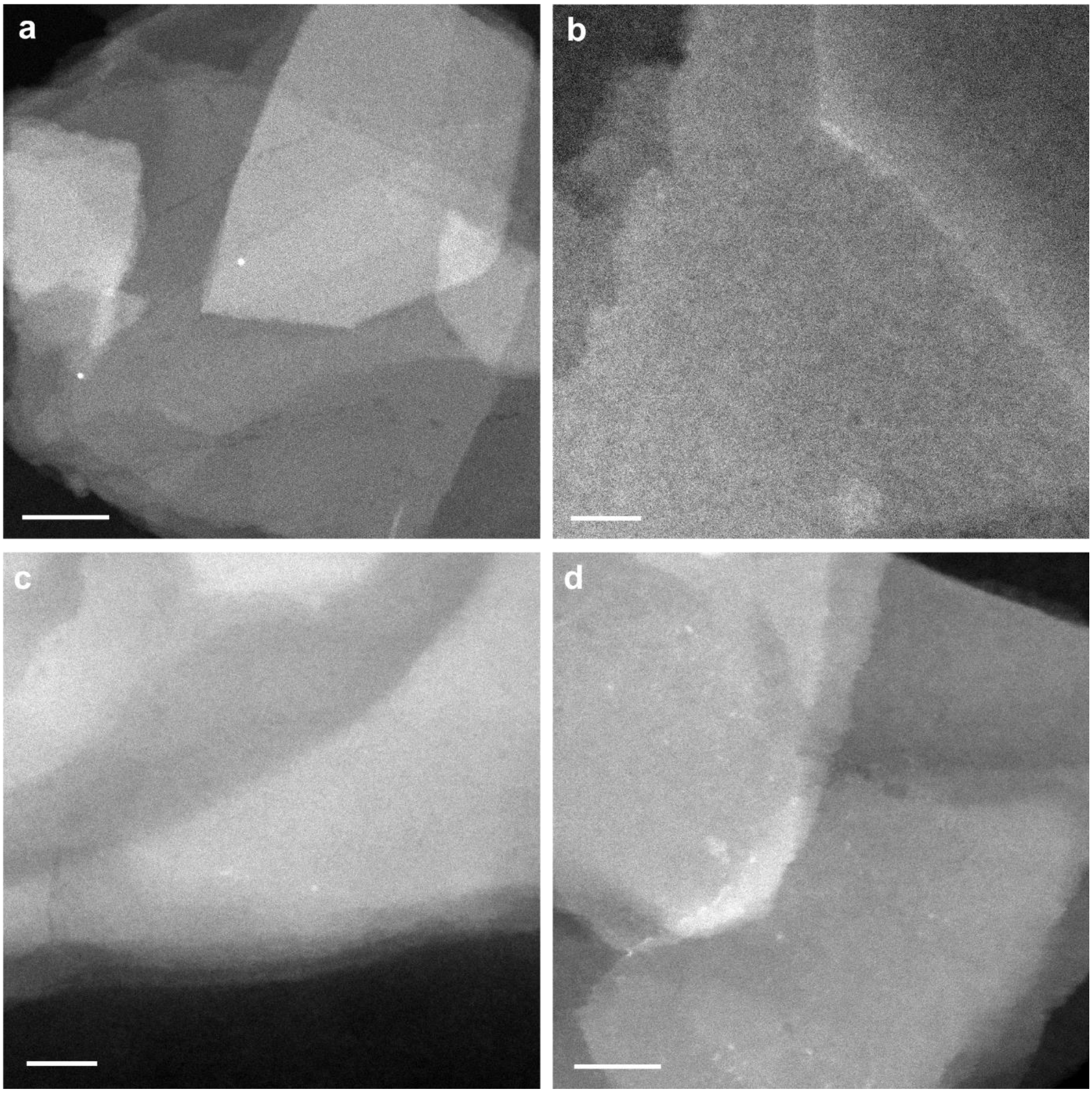

Supplementary Fig. 6 Morphological characterization of 0.17%Pt@MCM-22 sample after reduction by H_2 at 400 °C and another calcination treatment in air at 550 °C. It is clearly shown in the images (a-d) that, most of the small Pt nanoparticles in the sample were redispersed into Pt clusters and highly dispersed Pt species. Scale bar: (a) 50 nm, (b-d) 20 nm.

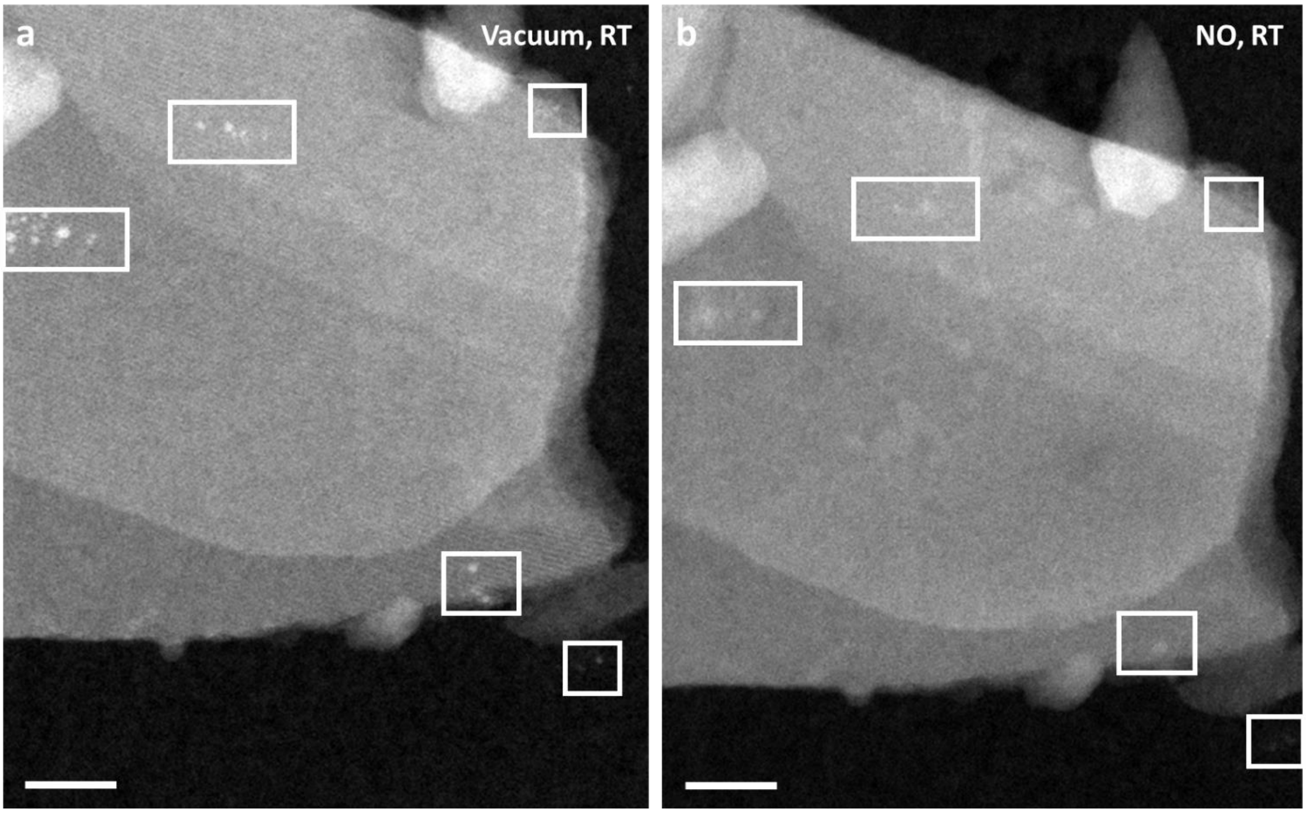

Supplementary Fig. 7 Evolution of Pt clusters in the presence of NO at room temperature. (a) In vacuum, Pt clusters around 0.8 nm are visible. (b) After introduction of NO (0.1 torr) and being kept in NO (0.1 torr) for 10 min, Pt clusters react with NO and show morphological transformation, corresponding to redispersion of Pt clusters into highly dispersed Pt species. In (a), both Pt clusters and nanoparticles are present. After treatment with NO at room temperature (b), Pt clusters (<1 nm) disappear and Pt nanoparticles (>1 nm) can still be observed, although the geometric shape of those Pt nanoparticles already change. Scale bar: (a, b) 20 nm.

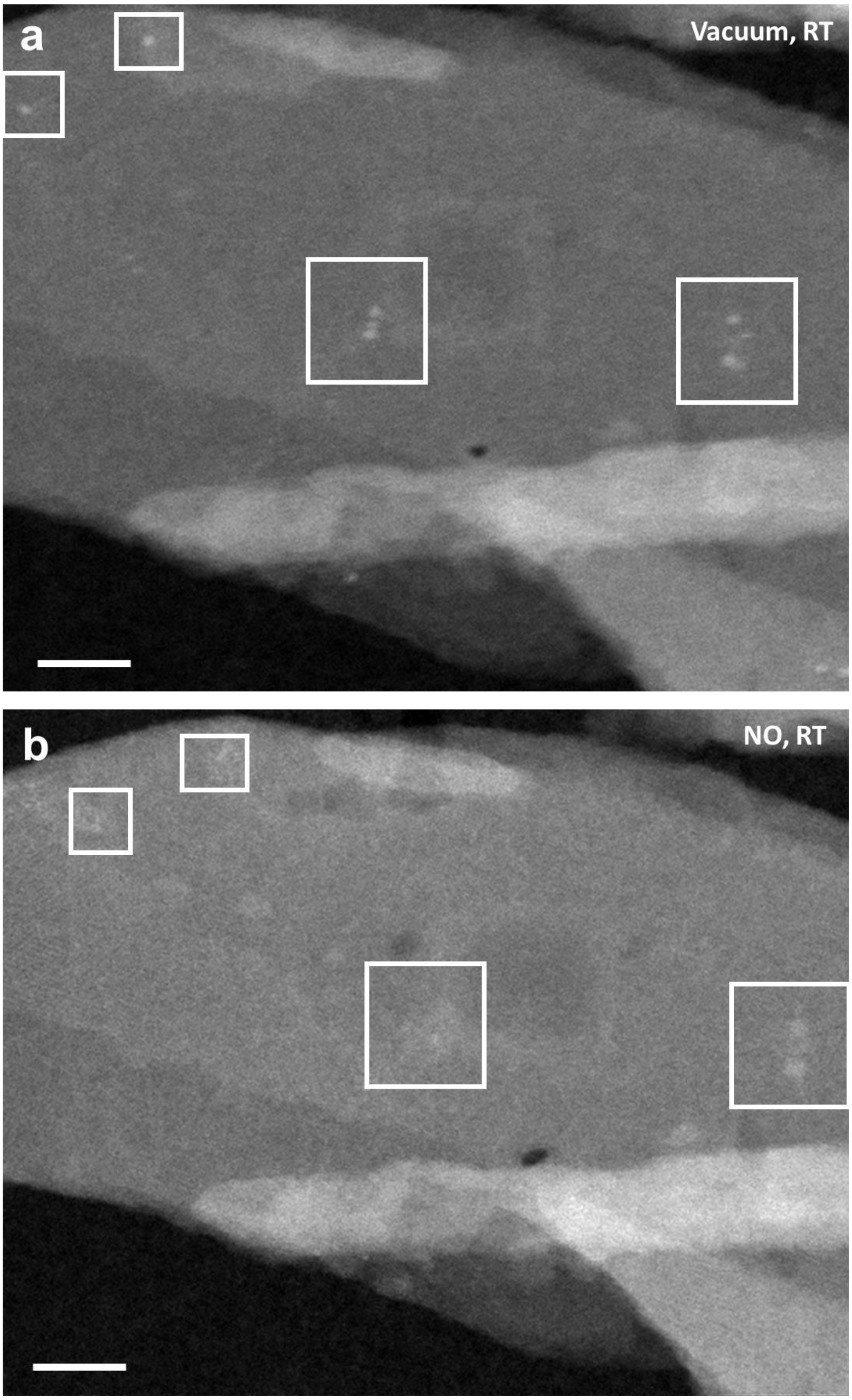

Supplementary Fig. 8 Evolution of Pt clusters in the presence of NO at room temperature. (a) STEM image of Pt particles in vacuum at room temperature. (b) STEM image of the same area after NO treatment at room temperature. The structural transformation of the Pt species is indicated by white rectangular. In (a), both Pt clusters and nanoparticles are present. After treatment with NO at room temperature (b), Pt clusters (<1 nm) disappear and Pt nanoparticles (>1 nm) can still be observed, although the geometric shape of those Pt nanoparticles already change. Scale bar: (a, b) 20 nm.

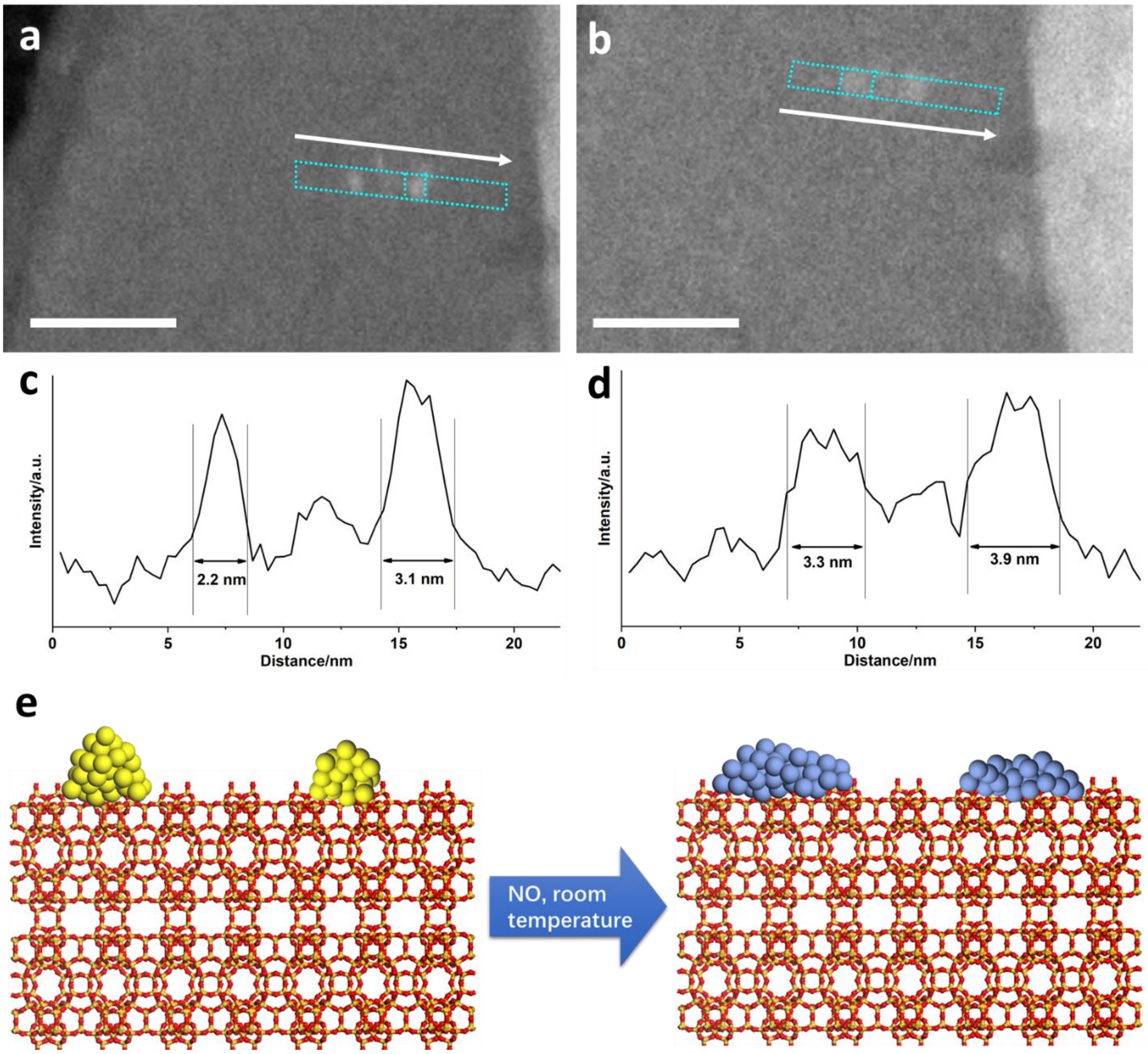

Supplementary Fig. 9 Structural transformation of Pt particles in NO. (a) STEM image of two Pt particles in vacuum. (b) STEM image of the same area after NO treatment at room temperature. (c) Intensity profile of the two Pt particles and their particle sizes. (d) Intensity profile of the two Pt particles and their particle sizes after NO treatment at room temperature. (e) Schematic illustration of structural transformation of Pt particles during the NO treatment at room temperature. Pt particles are oxidized by NO and the diameter of the particle also increases after the NO treatment. Scale bar: (a, b) 20 nm.

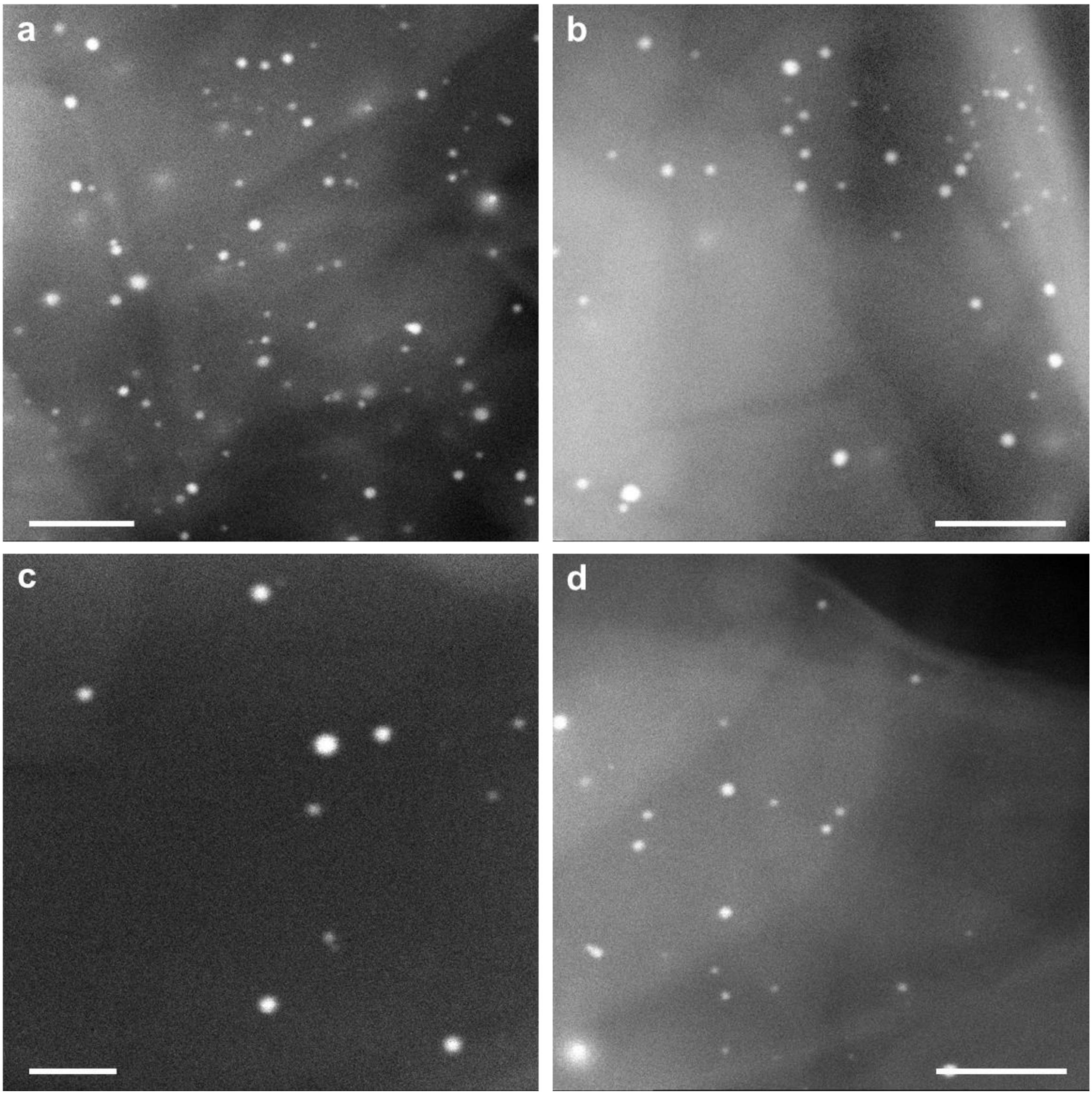

Supplementary Fig. 10 Morphological characterization of as-prepared 0.3%Pt@MCM-22 sample. As shown in the images (a-d), a large fraction of Pt nanoparticles is present in this sample, especially on the external surface of MCM-22 zeolite. Scale bar: (a, b, d) 50 nm, (c) 20 nm.

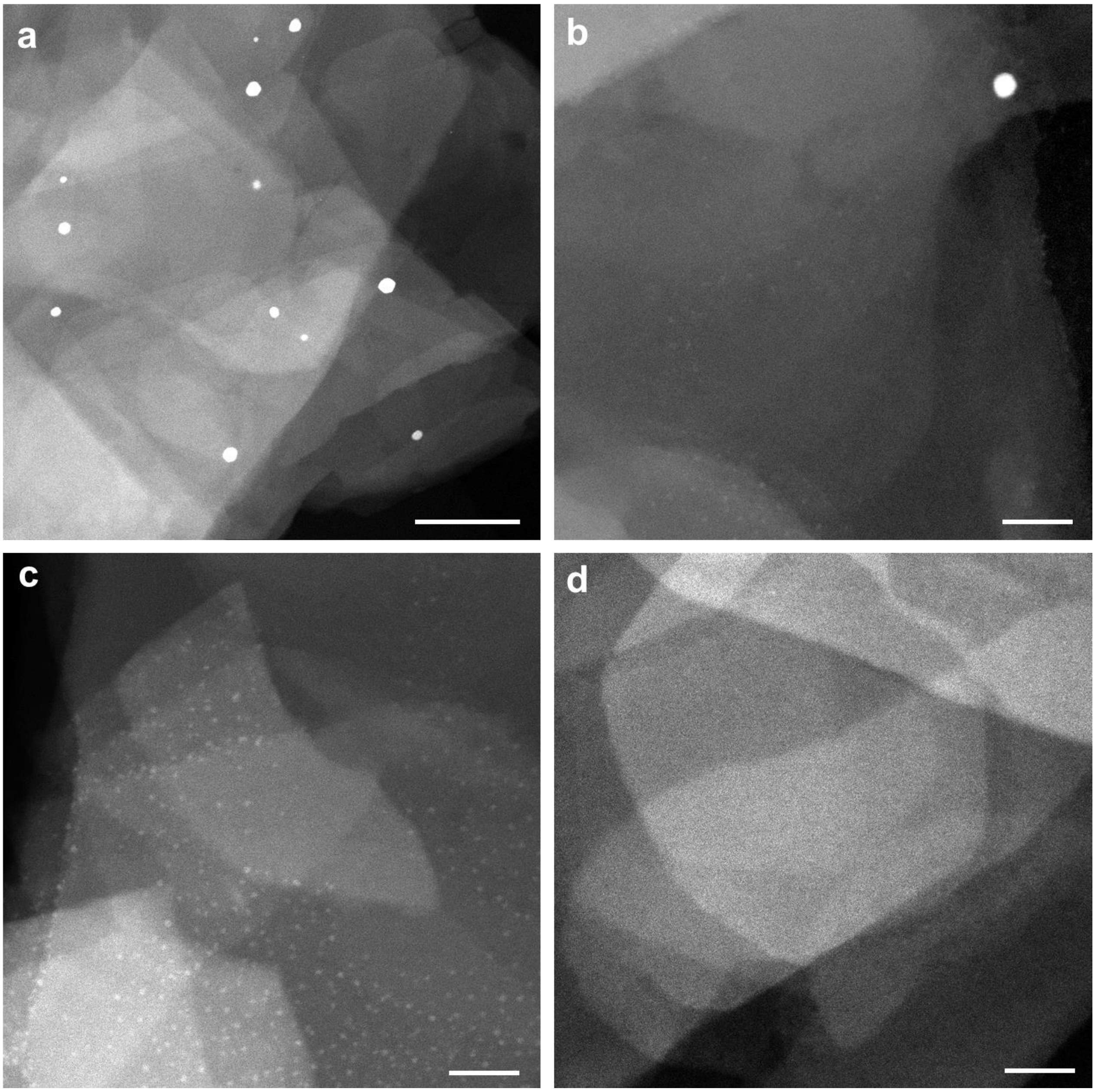

Supplementary Fig. 11 Morphological characterization of 0.3%Pt@MCM-22 sample after calcination in NO at 200 ºC. (a) After NO treatment at 200 ºC, it can be observed that, the number of the Pt nanoparticles decreases compared with the pristine 0.3%Pt@MCM-22 sample. (b-d) The STEM images show the presence of smaller Pt nanoparticles (1~2 nm) and subnanometric Pt clusters in this sample. Nevertheless, some Pt nanoparticles can still be observed on the surface of MCM-22 zeolite, suggesting only part of the Pt nanoparticles are redispersed during the NO treatment at 200 ºC. Scale bar: (a) 100 nm, (b-d) 20 nm.

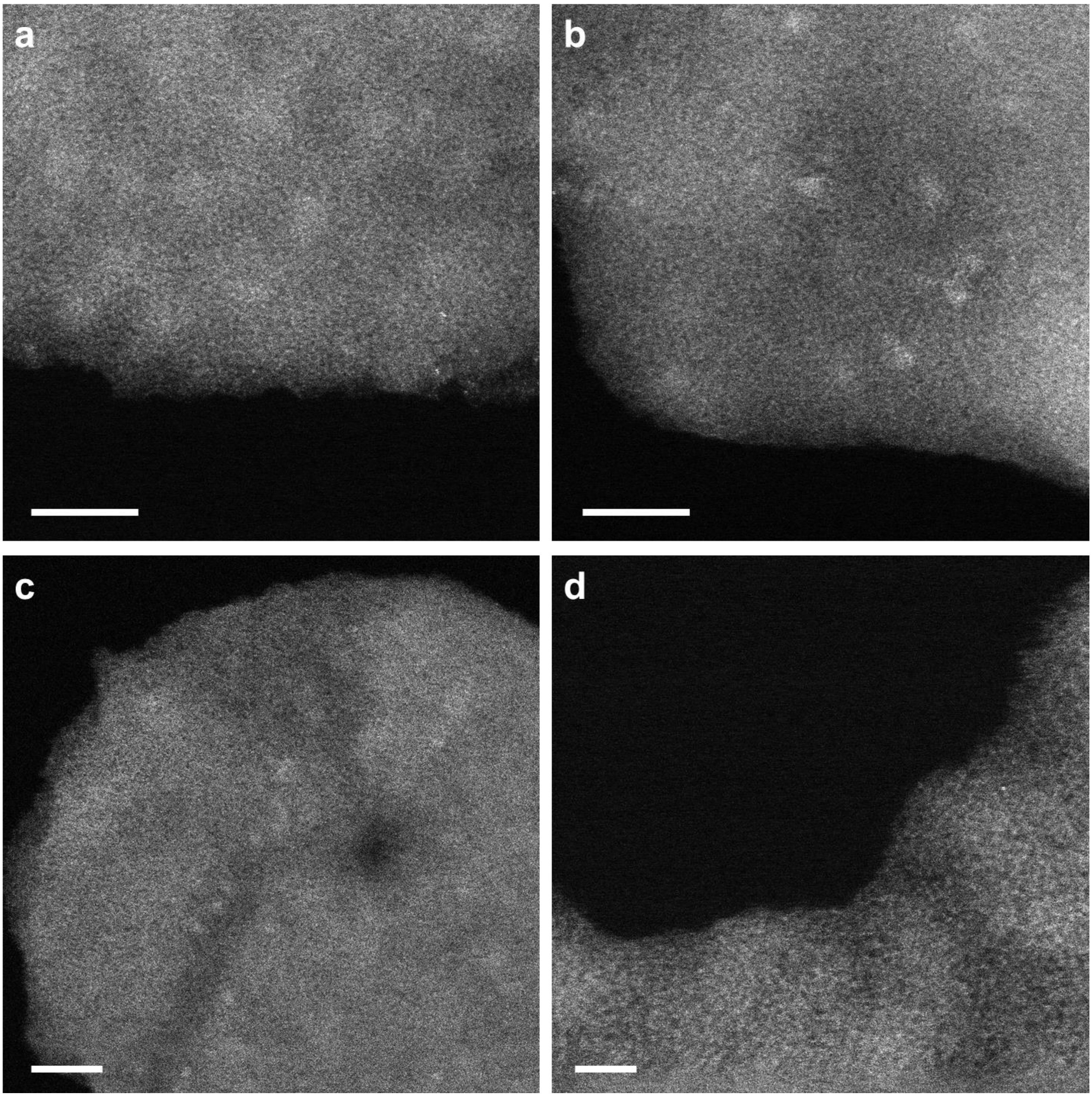

Supplementary Fig. 12 High-resolution STEM images of 0.3%Pt@MCM-22 sample after calcination in NO at 200 ºC. In these images (a-d), the presence of Pt clusters and singly dispersed Pt atoms can be clearly seen. These subnanometric Pt species should come from the redispersion of Pt nanoparticles in the pristine sample after NO treatment at 200 ºC. Scale bar: (a-c) 5 nm, (d) 2 nm.

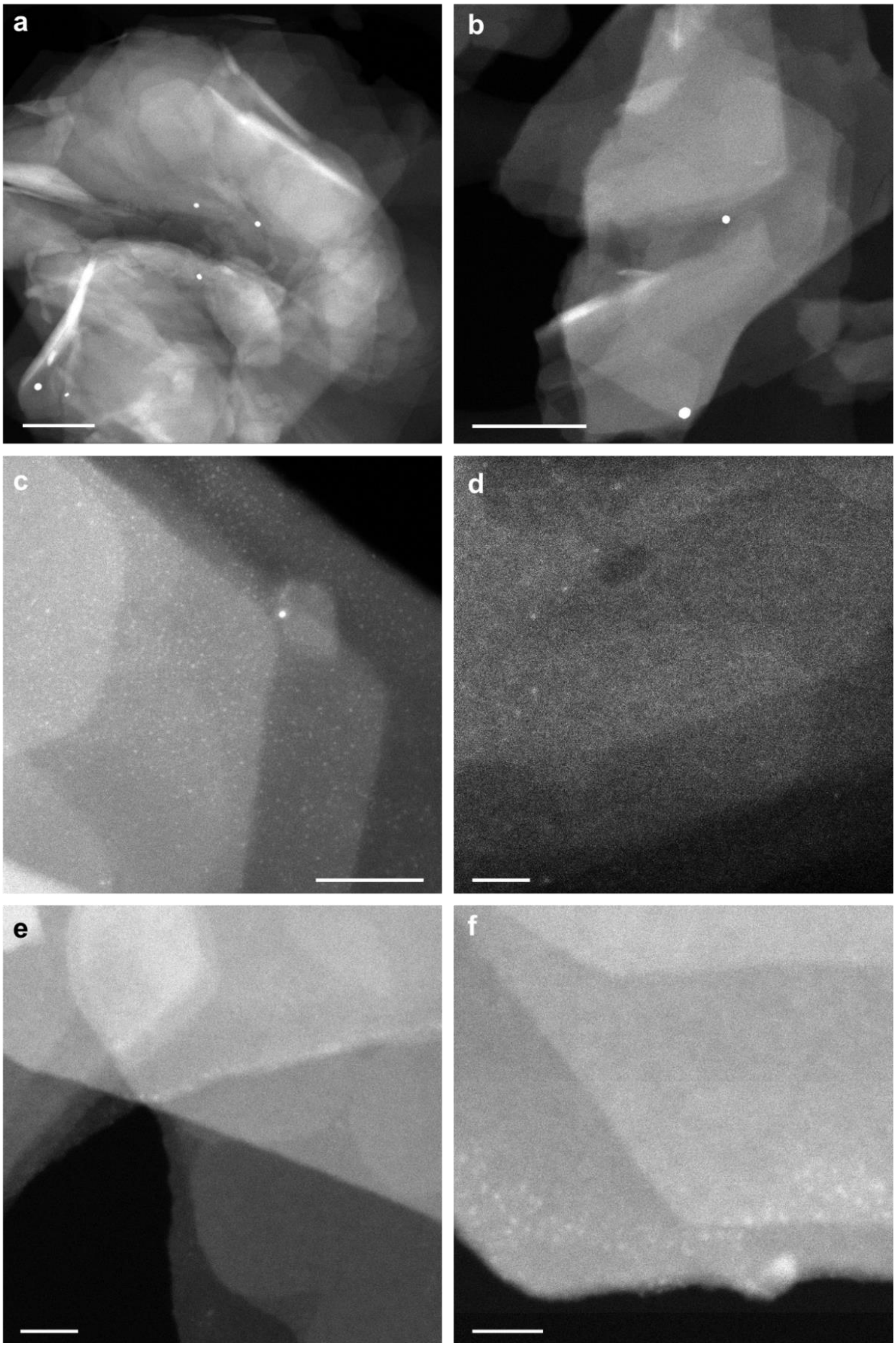

Supplementary Fig. 13 Morphological characterization of 0.3%Pt@MCM-22 sample after calcination in NO at 300 ºC. (a, b) As shown in these images, the number of Pt nanoparticles decrease significantly after NO treatment at 300 ºC compared to the sample after NO treatment at 200 ºC (shown in **Supplementary Fig. 11**). (c-d) Most of the Pt nanoparticles are re-dispersed, forming highly dispersed Pt species and subnanometric Pt clusters. Scale bar: (a, b) 200 nm, (c) 50 nm, (d-f) 20 nm.

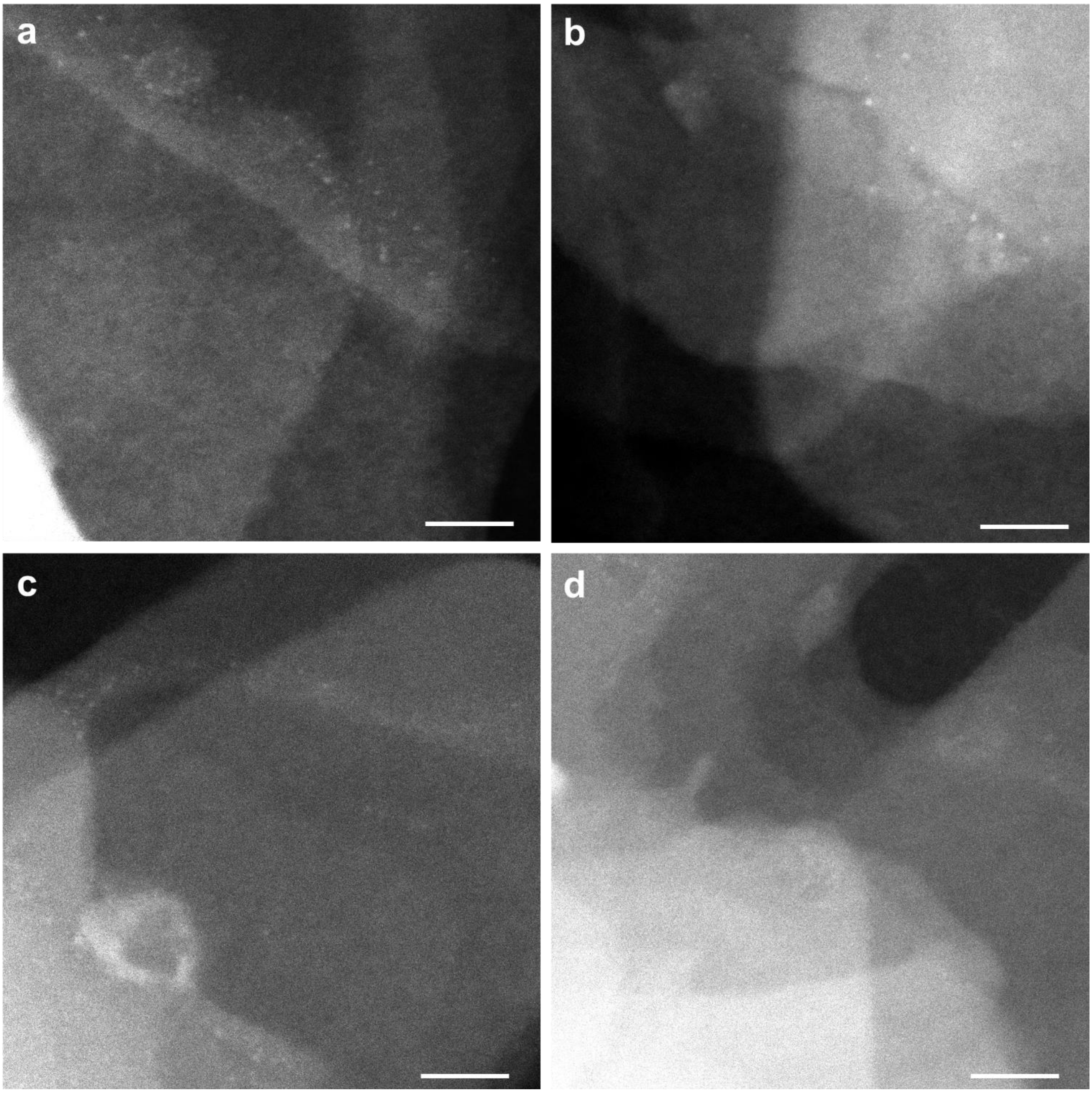

Supplementary Fig. 14 Morphological characterization of 0.3%Pt@MCM-22 sample after calcination in NO at 300 ^{o}C and subsequent reduction by H_2 at 200 ^{o}C. (a-d) As shown in the above STEM images, the size of Pt species are still remaining small (<2 nm) after the H_2 reduction treatment, suggesting the stability of the redispersed Pt species after NO treatment. Scale bar: (a-d) 20 nm.

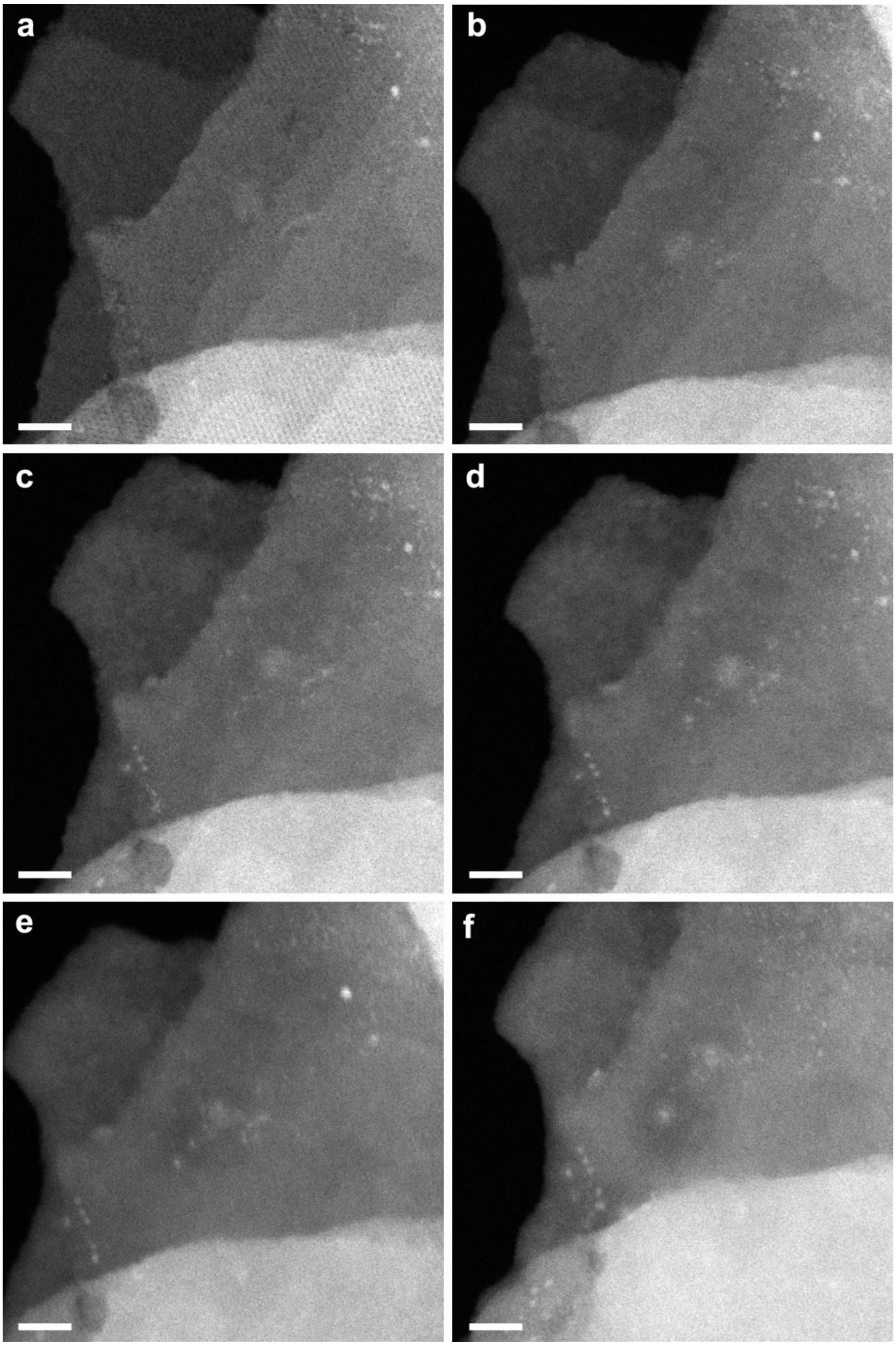

Supplementary Fig. 15 Structural evolution of 0.17%Pt@MCM-22-300H_2 catalyst under CO+O_2 reaction conditions at different reaction temperature measured by *in situ* TEM. During the *in situ* TEM experiments, the sample was treated in mixture of CO (0.2 torr) and O_2 (0.1 torr) for 15 min at different reaction temperature, respectively. In order to avoid the carbon deposition of CO by the electron beam, the TEM chamber was evacuated to remove CO gas after exposure of the sample to CO+O_2 gases at each temperature. (a) room temperature, (b) 100 ºC, (c) 150 ºC, (d) 200 ºC, (e) 300 ºC, (f) 400 ºC. Scale bar: (a-f) 10 nm.

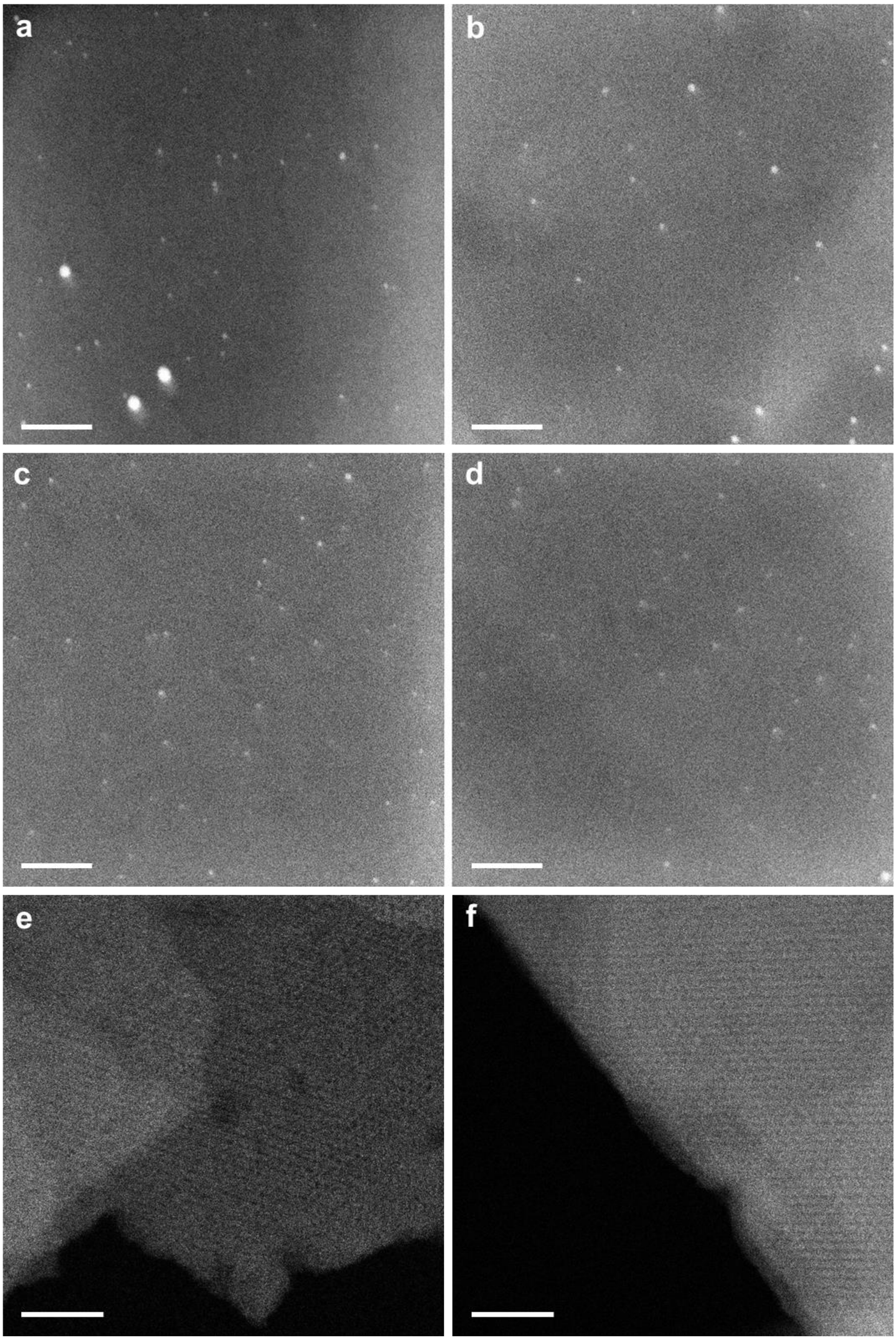

Supplementary Fig. 16 STEM images of 0.17Pt@MCM-22 sample after CO+O_2 reaction at 300 ^{o}C. Before the reaction, the pristine catalyst mainly contains highly dispersed Pt species, as shown in **Supplementary Figure 2**. (a-d) After the CO+O_2 reaction, Pt clusters and nanoparticles can be seen in the used catalyst. (e, f) The pore structures of MCM-22 can be seen In the high-resolution STEM images. Scale bar: (a-d) 20 nm, (e, f) 10 nm.

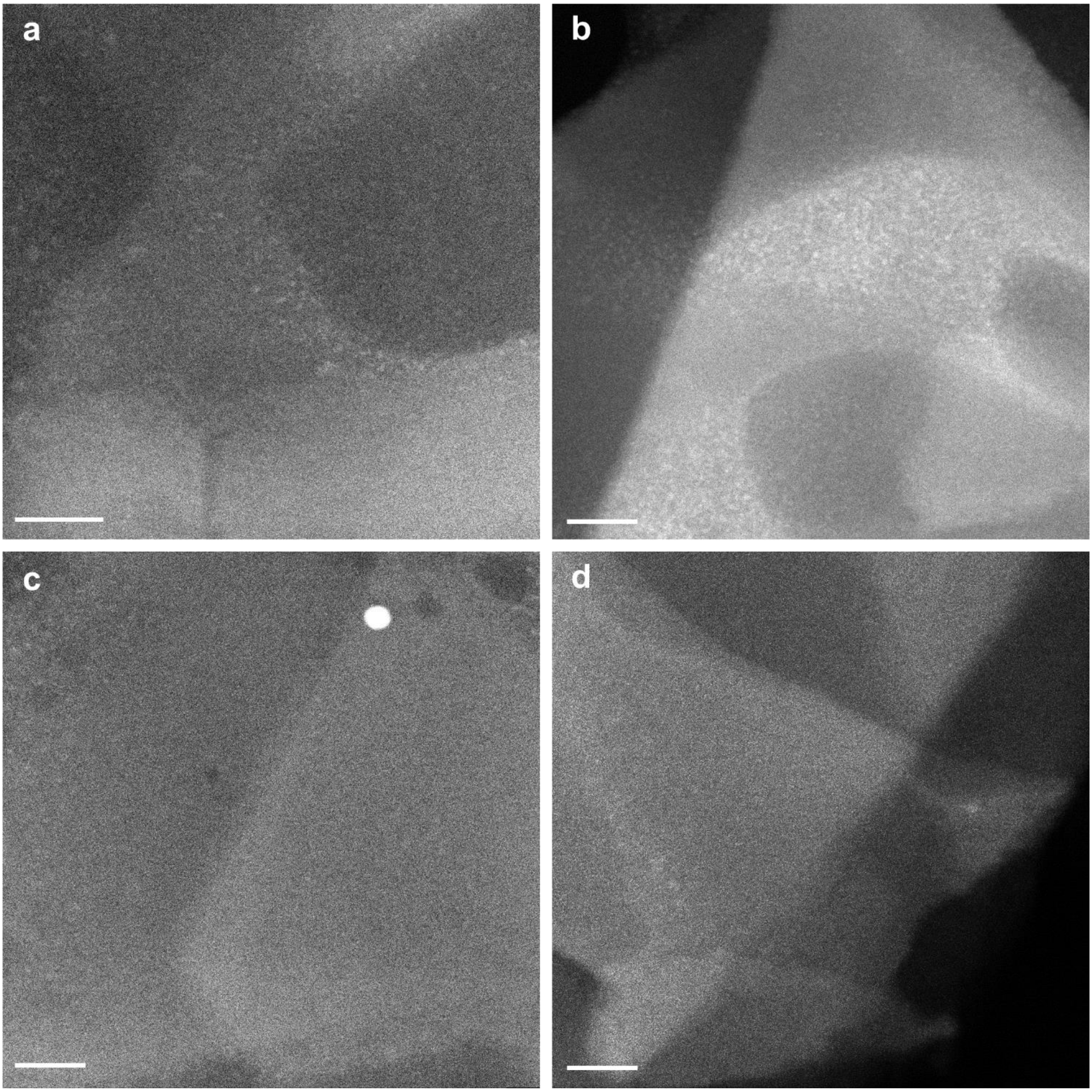

Supplementary Fig. 17 STEM images of 0.17Pt@MCM-22 sample after $CO+O_2$ reaction at 400 ºC. Before the reaction, the pristine catalyst mainly contains highly dispersed Pt species, as shown in **Supplementary Fig. 2**. After the $CO+O_2$ reaction at 300 ºC, Pt clusters and nanoparticles can be seen in the used catalyst. Afterwards, as shown in this figure (a-d), the reaction temperature is further elevated to 400 ºC and Pt nanoparticles become redispersed after the high-temperature treatment under $CO+O_2$ reaction conditions. Pt species mainly exist as clusters. A few Pt nanoparticles can be observed. Scale bar: (a-d) 20 nm.

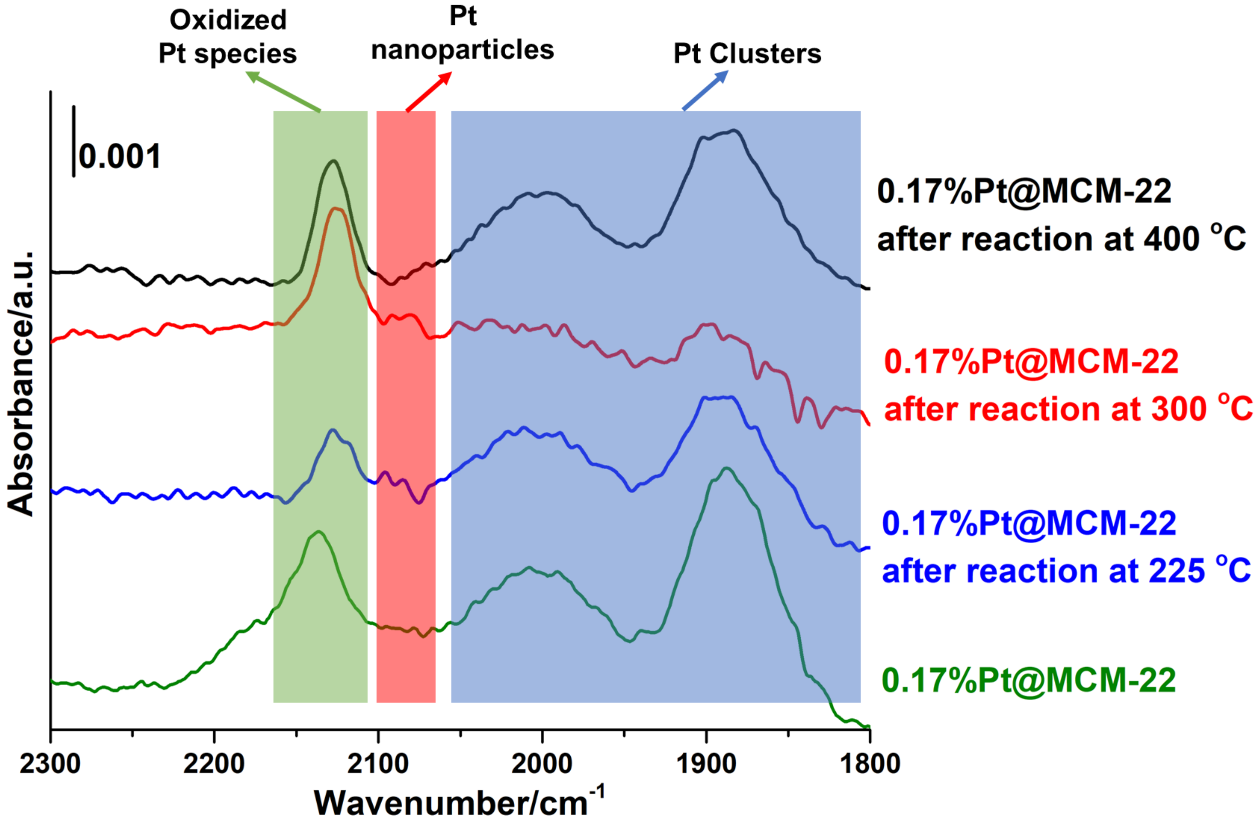

Supplementary Fig. 18 *In situ* CO-IR spectra of fresh 0.17%Pt@MCM-22 sample and the sample after $CO+O_2$ reaction at different temperature.

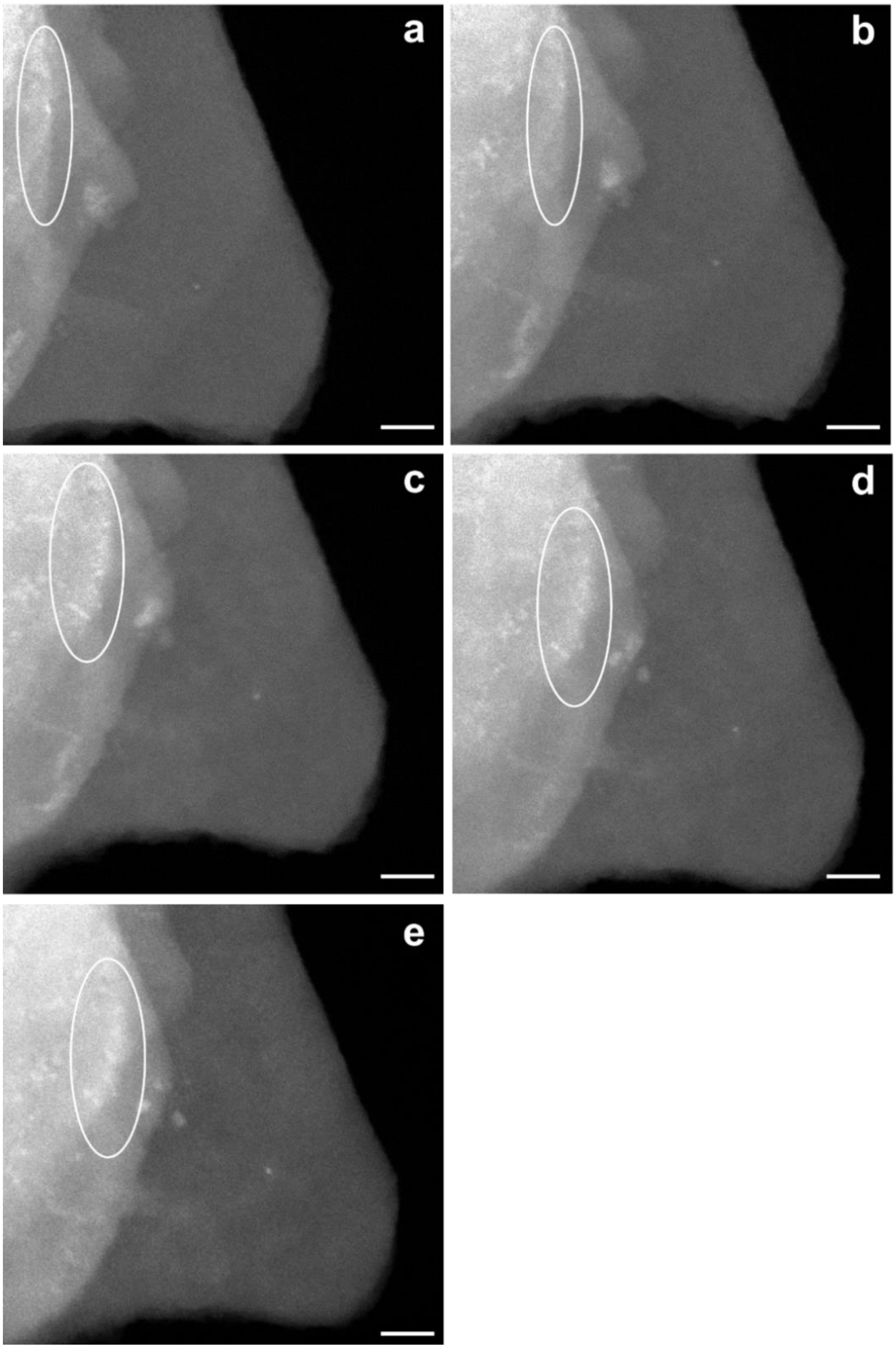

Supplementary Fig. 19 Evolution of Pt species in 0.17%Pt@MCM-22-300H$_2$ sample under water-gas shift (WGS) reaction conditions measured by *in situ* TEM. During the *in situ* TEM experiments, the sample was treated in mixture of CO (0.1 torr) and H_2O (0.2 torr) for 15 min at different reaction temperature, respectively. In order to avoid the carbon deposition of CO by the electron beam, the TEM chamber was evacuated to remove CO gas after exposure of the sample to CO+H_2O gases at each temperature. (a) room temperature, (b) 100 ºC, (c) 200 ºC, (d) 300 ºC and (e) 400 ºC. Scale bar in all the images in this figure: 10 nm. As indicated by the white circle, the agglomeration of highly dispersed Pt species into Pt clusters can be observed during the elevation of temperature. And those Pt clusters remain stable up to 400 ºC without redispersion. Scale bar in all the images in this figure: 10 nm.

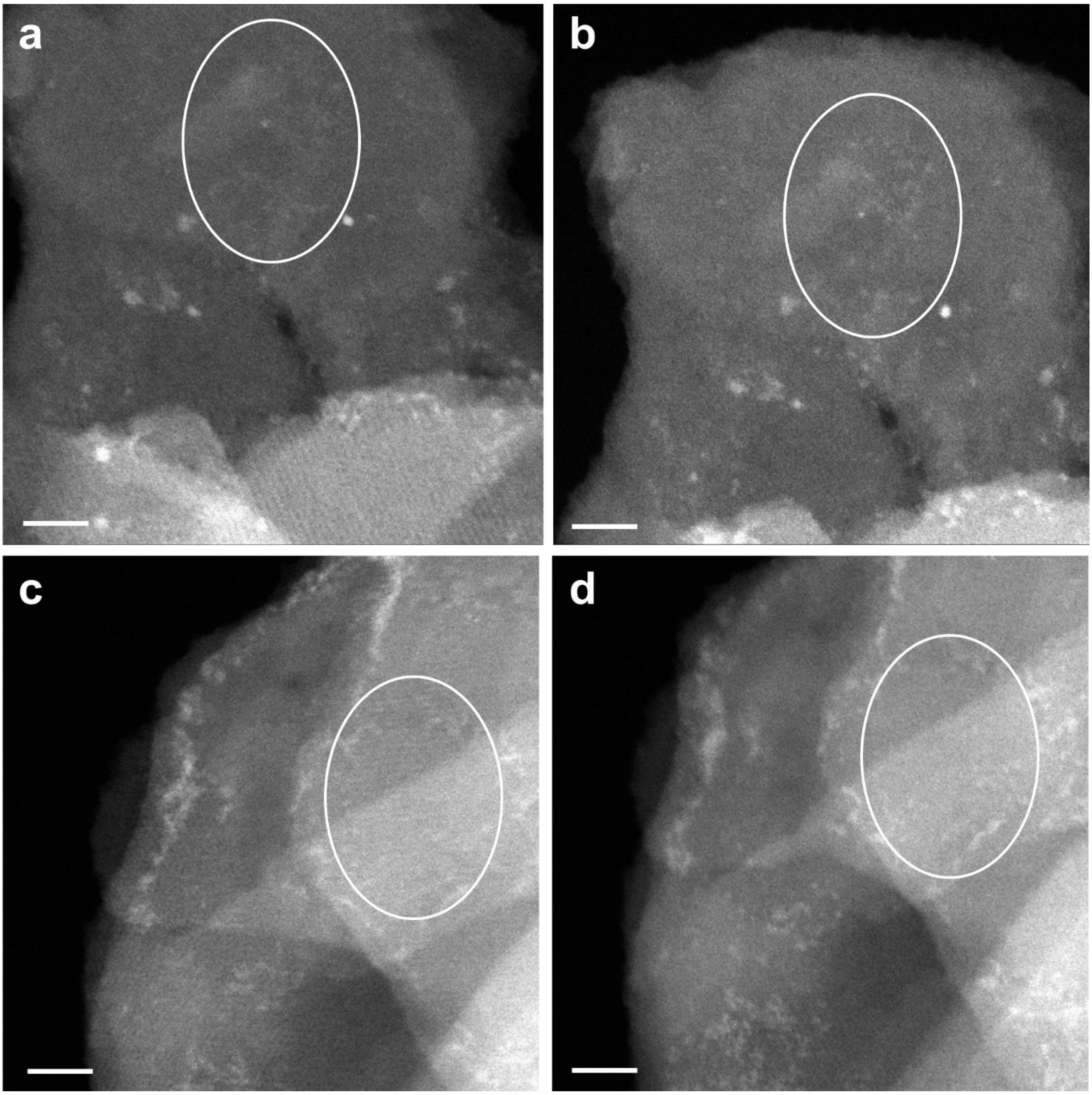

Supplementary Fig. 20 Evolution of Pt species in 0.17%Pt@MCM-22-300H$_2$ sample under water-gas shift (WGS) reaction conditions measured by *in situ* TEM in two different areas. (a, c) STEM image obtained at room temperature and (b, d) STEM images obtained at 100 ºC. During the *in situ* TEM experiments, the sample was treated in mixture of CO (0.1 torr) and H_2O (0.2 torr) for 15 min at different reaction temperature, respectively. In order to avoid the carbon deposition of CO by the electron beam, the TEM chamber was evacuated to remove CO gas after exposure of the sample to CO+H_2O gases at each temperature. The agglomeration of highly dispersed Pt species into Pt clusters are indicated by white circles. Scale bar in all the images in this figure: 10 nm.

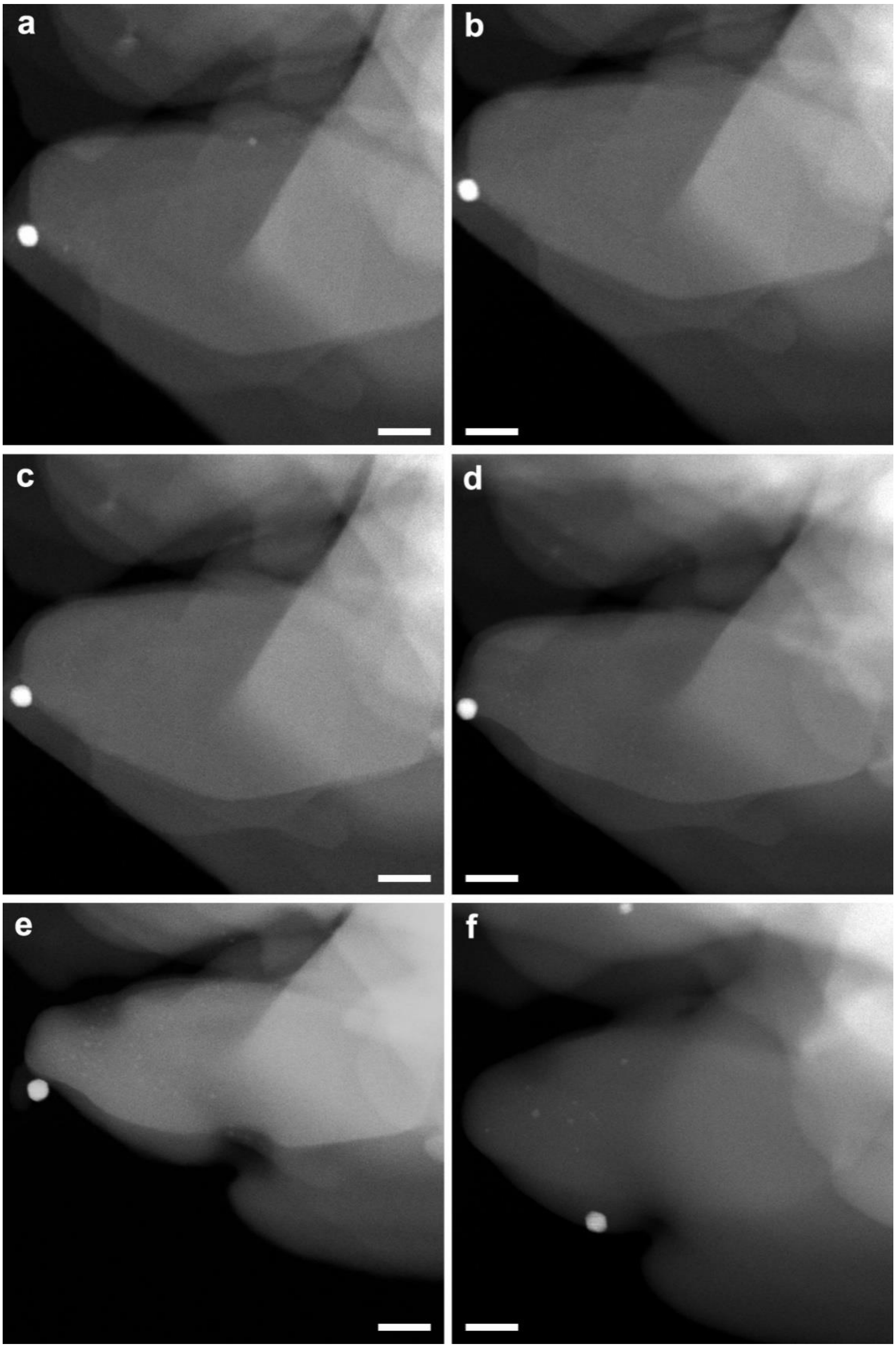

Supplementary Fig. 21 High-temperature stability test of 0.17%Pt@MCM-22-300H_2 catalyst under NO+CO reaction conditions measured by *in situ* TEM. During the in situ TEM experiments, the sample was treated in mixture of CO (0.1 torr) and NO (0.1 torr) for 15 min at different reaction temperature, respectively. In order to avoid the carbon deposition of CO by the electron beam, the TEM chamber was evacuated to remove CO gas after exposure of the sample to CO+NO gases at each temperature. (a) 200 ºC, (b) 400 ºC, (c) 600 ºC, (d) 800 ºC, (e) 1000 ºC, (f) 1200 ºC. Scale bar in all the images in this figure: 20 nm.

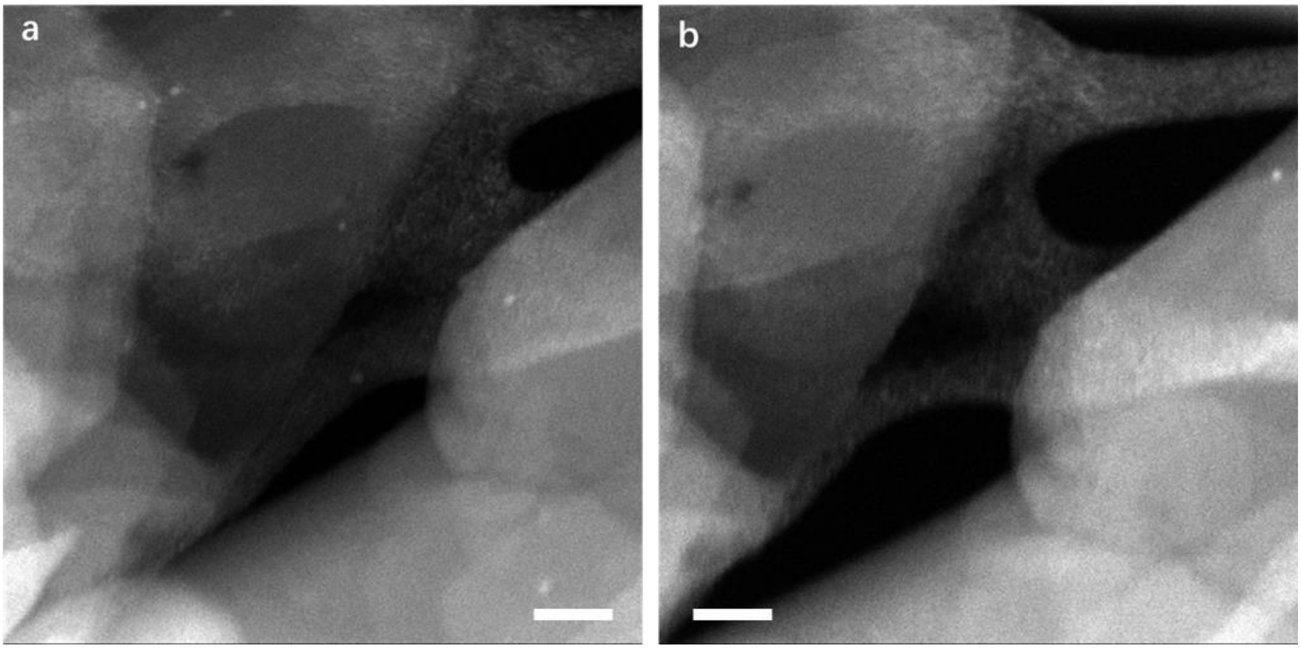

Supplementary Fig. 22 Evolution of Pt clusters from 200 ºC to 400 ºC in NO+CO atmosphere. As shown in the STEM image, Pt clusters were observed at 200 ºC (a) and they disappeared when the temperature was increased to 400 ºC (b). Scale bar: (a, b) 20 nm.

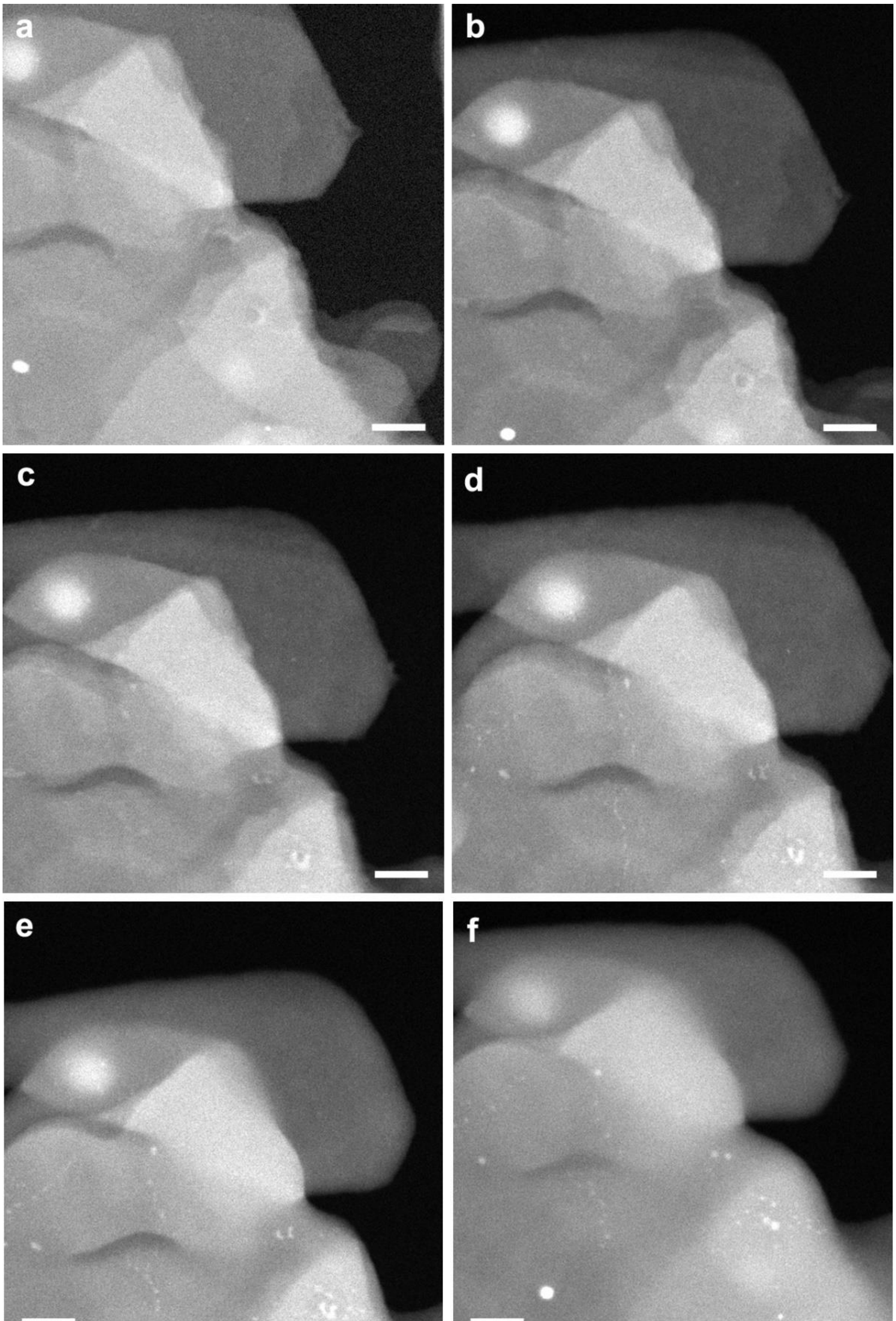

Supplementary Fig. 23 High-temperature stability test of 0.17%Pt@MCM-22-300H_2 catalyst under H_2+NO reaction conditions measured by *in situ* TEM. During the in situ TEM experiments, the sample was treated in mixture of H_2 (0.1 torr) and NO (0.1 torr) for 15 min at different reaction temperature, respectively. The above images were recorded in the presence of NO+H_2 gases at different temperature. (a) 200 ºC, (b) 400 ºC, (c) 600 ºC, (d) 800 ºC, (e) 1000 ºC, (f) 1200 ºC. Scale bar in all the images in this figure: 20 nm.

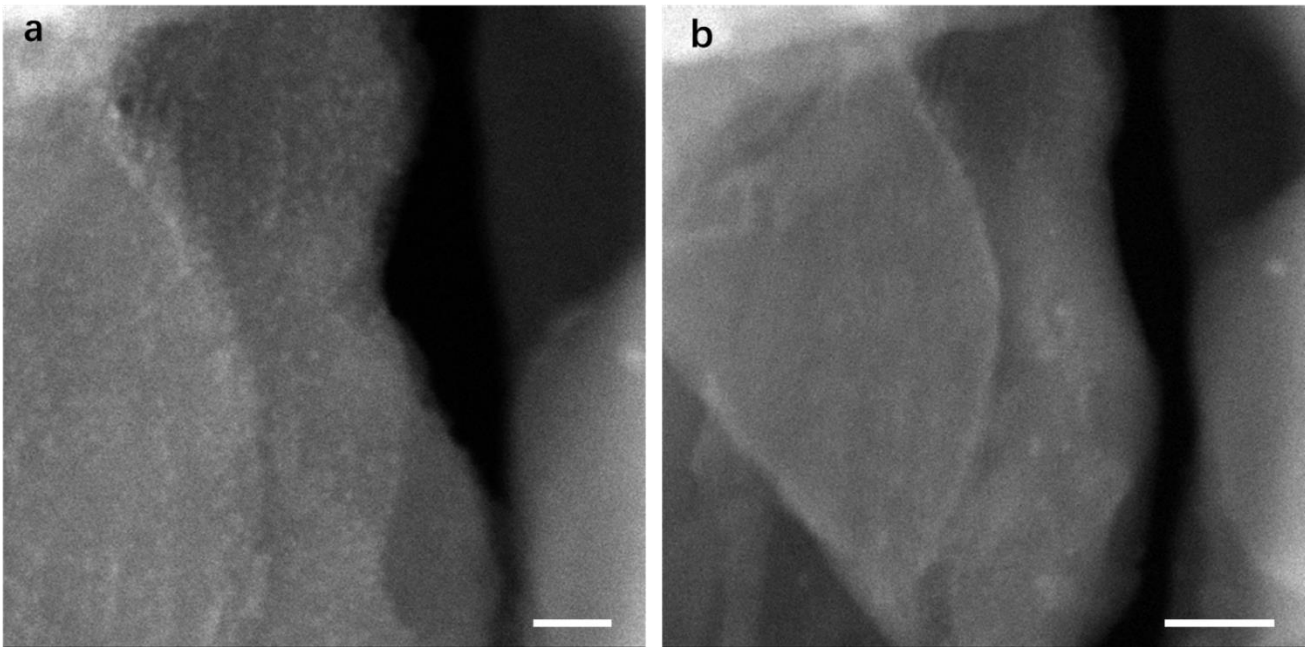

Supplementary Fig. 24 Evolution of Pt clusters from room temperature to 200 °C in $NO+H_2$ atmosphere. As shown in the STEM image, Pt clusters were observed at room temperature (a) and most of them disappeared when the temperature was increased to 200 °C (b). Scale bar: (a) 10 nm, (b) 20 nm.
